# Intrinsic spin shielding effect in platinum–rare-earth alloy boosts oxygen reduction activity

**DOI:** 10.1093/nsr/nwad162

**Published:** 2023-06-09

**Authors:** Siyuan Zhu, Mingzi Sun, Bingbao Mei, Liting Yang, Yuyi Chu, Zhaoping Shi, Jingsen Bai, Xian Wang, Zheng Jiang, Changpeng Liu, Bolong Huang, Junjie Ge, Wei Xing

**Affiliations:** State Key Laboratory of Electroanalytical Chemistry, Laboratory of Advanced Power Sources, Changchun Institute of Applied Chemistry, Chinese Academy of Sciences, Changchun130022, China; School of Applied Chemistry and Engineering, University of Science and Technology of China, Hefei230026, China; Department of Applied Biology and Chemical Technology, The Hong Kong Polytechnic University, Hong Kong, China; Shanghai Synchrotron Radiation Facility, Zhangjiang National Laboratory, Shanghai Advanced Research Institute, Chinese Academy of Sciences, Shanghai201204, China; State Key Laboratory of Electroanalytical Chemistry, Laboratory of Advanced Power Sources, Changchun Institute of Applied Chemistry, Chinese Academy of Sciences, Changchun130022, China; School of Applied Chemistry and Engineering, University of Science and Technology of China, Hefei230026, China; State Key Laboratory of Electroanalytical Chemistry, Laboratory of Advanced Power Sources, Changchun Institute of Applied Chemistry, Chinese Academy of Sciences, Changchun130022, China; School of Applied Chemistry and Engineering, University of Science and Technology of China, Hefei230026, China; State Key Laboratory of Electroanalytical Chemistry, Laboratory of Advanced Power Sources, Changchun Institute of Applied Chemistry, Chinese Academy of Sciences, Changchun130022, China; School of Applied Chemistry and Engineering, University of Science and Technology of China, Hefei230026, China; State Key Laboratory of Electroanalytical Chemistry, Laboratory of Advanced Power Sources, Changchun Institute of Applied Chemistry, Chinese Academy of Sciences, Changchun130022, China; School of Applied Chemistry and Engineering, University of Science and Technology of China, Hefei230026, China; State Key Laboratory of Electroanalytical Chemistry, Laboratory of Advanced Power Sources, Changchun Institute of Applied Chemistry, Chinese Academy of Sciences, Changchun130022, China; School of Applied Chemistry and Engineering, University of Science and Technology of China, Hefei230026, China; Shanghai Synchrotron Radiation Facility, Zhangjiang National Laboratory, Shanghai Advanced Research Institute, Chinese Academy of Sciences, Shanghai201204, China; State Key Laboratory of Electroanalytical Chemistry, Laboratory of Advanced Power Sources, Changchun Institute of Applied Chemistry, Chinese Academy of Sciences, Changchun130022, China; School of Applied Chemistry and Engineering, University of Science and Technology of China, Hefei230026, China; Department of Applied Biology and Chemical Technology, The Hong Kong Polytechnic University, Hong Kong, China; State Key Laboratory of Electroanalytical Chemistry, Laboratory of Advanced Power Sources, Changchun Institute of Applied Chemistry, Chinese Academy of Sciences, Changchun130022, China; School of Applied Chemistry and Engineering, University of Science and Technology of China, Hefei230026, China; Dalian National Laboratory for Clean Energy, Chinese Academy of Sciences, Dalian116023, China; State Key Laboratory of Electroanalytical Chemistry, Laboratory of Advanced Power Sources, Changchun Institute of Applied Chemistry, Chinese Academy of Sciences, Changchun130022, China; School of Applied Chemistry and Engineering, University of Science and Technology of China, Hefei230026, China

**Keywords:** oxygen reduction reaction, intermetallic compound, spin effect, rare-earth metal, electrocatalysis

## Abstract

Oxygen reduction reactions (ORRs) involve a multistep proton-coupled electron process accompanied by the conversion of the apodictic spin configuration. Understanding the role of spin configurations of metals in the adsorption and desorption of oxygen intermediates during ORRs is critical for the design of efficient ORR catalysts. Herein, a platinum–rare-earth-metal-based alloy catalyst, Pt_2_Gd, is introduced to reveal the role of spin configurations in the catalytic activity of materials. The catalyst exhibits a unique intrinsic spin reconfiguration because of interactions between the Gd-4f and Pt-5d orbitals. The adsorption and desorption of the oxygen species are optimized by modifying the spin symmetry and electronic structures of the material for increased ORR efficiency. The Pt_2_Gd alloy exhibits a half-wave potential of 0.95 V and a superior mass activity of 1.5 A·mg_Pt_^−1^ in a 0.1 M HClO_4_ electrolyte, as well as higher durability than conventional Pt/C catalysts. Theoretical calculations have proven that the spin shielding effect of Gd pairs increases the spin symmetry of Pt-5d orbitals and adsorption preferences toward spin-polarized intermediates to facilitate ORR. This work clarifies the impact of modulating the intrinsic spin state of Pt through the interaction with the local high spin 4f orbital electrons in rare-earth metals, with the aim of boosting the spin-related oxygen reduction reaction, thus fundamentally contributing to the understanding of new descriptors that control ORR activity.

## Teaser

Deliberately constructing the model for manipulating ORR performance of spin polarized catalysts and accurately decoupling the relationship between the spin state in spin reconfigured alloy and the adsorption strength of the oxide intermediate species.

## INTRODUCTION

Proton exchange membrane fuel cells (PEMFCs) are promising energy supply devices, and the electrocatalysis efficiency of the reaction between the fuel and oxygen molecules at the electrodes largely determines the feasibility of PEMFCs [1–3]. The oxygen reduction reaction (ORR) at the cathode exhibits sluggish kinetics because it involves a slow and energy-intensive four-electron process, whereas anode reactions are rapid, which affects electronic mobility in the fuel cell [4,5]. Thus, platinum (Pt)-based cathode catalysts with satisfactory activity and durability have been developed [6,7], and general activity descriptors have been studied to reveal overall ORR performance [8–11].

During the ORR, the outermost d orbitals of Pt interact with the oxygen molecule, resulting in a 5d–2p orbital hybridization that produces a deep-lying filled bonding state and partially filled antibonding states. Therefore, the adsorption strength depends on the energy levels and filling state of the antibonding orbital. Alloying Pt with transitional metals (M) can decrease the energy levels of the antibonding orbitals while increasing the intrinsic activity of Pt, particularly in the harsh acidic reaction environment of the fuel cell. Non-structured PtM alloys can significantly increase ORR activity because of their crystal phase and tunable plane indices, which are achieved via a ligand or a strain effect to reduce the Pt d-band center and intermediate binding strength [3,6,12–15]. The d-band energy modulation has been widely and successfully used in most cases. However, emerging studies have been focused on investigating more profound Pt–M interactions, such as the occupation of d orbitals in Pt–Fe pair sites and quantum spin-exchange interactions in Pt_3_M [16,17].

Electrons are undoubtedly not only carriers of charge, but also carriers of spin [18]. In recent years, electronic asymmetry has been a promising approach to improving electroactivity. Yao *et al.* conducted a series of studies to achieve asymmetric electronic distributions near active sites, which accelerate electrocatalysis because of charge polarizations and an increase in delocalized electrons [19–22]. The spin effect is another method used to modulate the electroactivity of catalysts, by forming spin-selective conduction channels to enhance adsorption. Electronic asymmetry, which is commonly used in atomic catalysts, modulates local active sites to directly improve the electroactivity of materials. In contrast, the spin effect is mostly applied to naturally magnetic transition metal oxides or metal alloys, with the goal of improving intermediate adsorption across the entire electrocatalyst. These effects often coexist in several materials, allowing for the collective enhancement of their electroactivity. A previous study revealed that when the electronic asymmetry is identical, antiparallel spin states between O_2_ and the catalyst improve their binding; however, the mechanism of this process is still unclear [23]. Thus, theoretical and experimental studies are still needed to further explore this field.

The study of the orbital configurations of oxygen molecules using quantum spin theory reveals that the transition from a triplet oxygen molecule to a singlet H_2_O molecule involves a spin-state variation [18,24–26]. Theoretical calculations have revealed that only when localized spins and electron magnetic interplay are included can the structure–property relationship be systematically comprehended [27–32]. The transformation of the bulk phase of the electrocatalyst from ferromagnetic to non-ferromagnetic can result in an energy shift of at least 0.4 eV during the adsorption of oxygen species on the catalyst surface [33]. To this end, the spin-related interaction of localized and itinerant d electrons may have a significant effect on the electrocatalytic reaction, particularly in the ORR process that involves spin flipping. The spin effect, however, has not been understood as well as the strain effect in metal-based compounds [34,35], thereby resulting in confusion or even misunderstanding, and a need for further exploration and achieving even higher intrinsic activity towards the ORR. Based on this, we hypothesized that spin regulation of catalysts via smartly engaging dominant magnetism could allow us to modulate electron transfer and optimize adsorption strength for localized intermediates.

Gadolinium (Gd) has an intrinsically high spin density and a natural magnetic moment because all of its 4f orbitals are half filled. When Gd is alloyed with another metal component, its rich single electrons in the open shell can change the spin state and electron delocalization of the other metal via orbital coupling in alloys. In this regard, we have successfully engaged Gd into the Pt lattice and presented intrinsic spin-reconfigured Pt_2_Gd intermetallic alloy for fast ORR kinetics. The Pt_2_Gd alloy exhibited remarkable electroactivity, with a high mass activity of 1.5 A·mg_Pt_^−1^ and a half-wave potential of 0.95 V in the acidic electrolyte environment, as well as superior durability. 4f orbitals of Gd, which have an open shell structure, can configure unbalanced electron spins in electron clouds. This can affect the magnetic relaxation of neighboring Pt nuclei. Density functional theory (DFT) calculations were used to clarify the modulations of Pt-5d orbitals through the spin reconfiguration induced by alloying with Gd. Moreover, the d–d orbital complementation between Gd and Pt increases electron transfer at Pt surface sites. This unique interplay between Pt and Gd, as well as the spin shielding effect of Gd-4f orbitals, significantly reduces the energy barrier of the rate-determining step, which enhances the ORR performance of the catalyst. This study contributes significantly to the design of novel electrocatalysts based on the spin-modulation strategy.

## RESULTS AND DISCUSSION

### Design of the synthesis method for Pt_2_Gd alloy

**Figure 1.**
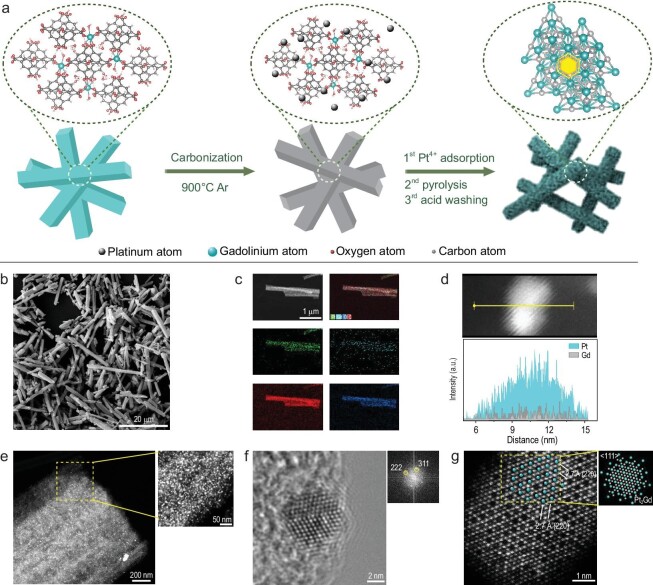
Synthesis presentation. (a) Schematic illustration of Pt_2_Gd-x alloy preparation. (b) Scanning electron microscope (SEM) image of the carbonized MOF-76(Gd). (c) STEM image and the corresponding EDS elemental mapping of Pt_2_Gd-900 alloy. (d) STEM-EDS line-scanning intensity profile (bottom) of a single nanoparticle. The top image shows the studied nanoparticle and the line-scanning analysis along the yellow line. (e) Representative annular dark-field STEM image of the Pt_2_Gd-900 supported on the Gd-MOF (inset: image from the partially enlarged view). (f) Atomic-resolution HR-TEM images (inset: fast Fourier transform (FFT) images obtained from the HR-TEM image). (g) High-angle annular dark-field images with inset simulated structure along ⟨111⟩ direction of Pt_2_Gd-900 alloy.

The standard reduction potentials of Pt and rare-earth metals are considerably different [36]. Thus, the synthesis of Pt–Gd alloy using the conventional wet chemical method is highly challenging [37]. Herein, we pioneered an atomically confined pyrolysis method to prepare the Pt–rare-earth alloy. Figure 1a shows the two main steps involved in this method. Considering that rare-earth metals are characterized by their high oxygen affinity, Gd ions were first anchored into the stable ordered framework of a metal–organic framework (MOF-76(Gd), i.e. [Gd(BTC)H_2_O]_n_, BTC: benzene–1,3,5-tricarboxylate), which is bonded to oxygen-containing ligand complexes (Fig. 1b). MOF-76(Gd) was subsequentially carbonized at 900°C to produce a substrate with confined crystalline growth. The substrate was then subjected to an impregnation process to introduce a Pt precursor, followed by a second thermal treatment at different temperatures in a mixed gas atmosphere of H_2_ and Ar. The MOF structure ensures atomic dispersion of the Gd species, which has a dominantly high chemical potential as well as metal oxide feature (Fig. S1) [38]. This facilitates the subsequent diffusion of Gd into Pt and the confinement of Pt by strong metal–support interactions in the form of small nanoparticles (NPs). The final catalysts are denoted Pt_2_Gd-x, where x represents the annealing temperature (700–1100°C).

To identify the formation of a bimetallic alloy, the morphology and components of the as-prepared samples were carefully analyzed. A representative alloy (Pt_2_Gd-900) was characterized to confirm the superior characteristics of the proposed alloys. Figure 1b–g clearly shows the formation of intermetallic Pt_2_Gd NPs on carbon nanorods in MOF-76(Gd). Annular dark-field scanning transmission electron microscopy (STEM) images and energy-dispersive spectroscopy (EDS) profiles show that the as-synthesized Pt_2_Gd-900 alloy NPs exhibit a starry permutation and narrow-range diameter distribution (Fig. 1c; Fig. 1e and its inset, which shows a partially enlarged image; Figs S3–S5). This proved that the atomic-assisted method efficiently prevented the formation of large quantities of Gd oxides, which have a low chemical potential [38]. The X-ray photoelectron spectroscopy (XPS) analysis further confirmed the presence of Pt and Gd in the catalyst. The XPS results revealed that the Pt/Gd atomic ratio is 0.31 : 0.14, which is consistent with inductively coupled plasma–optical emission spectroscopy (ICP–AES) measurements (mass ratio = 2.75 : 1.00).

**Figure 2.**
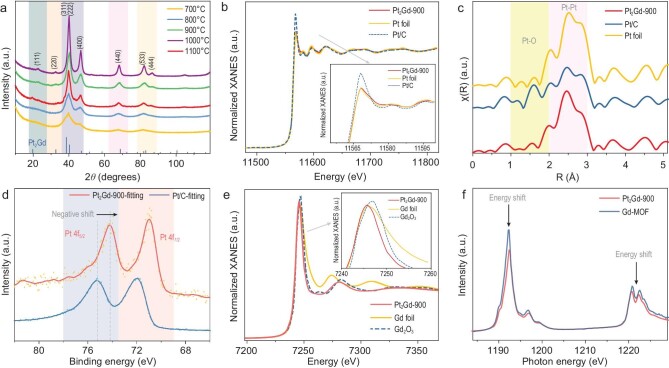
Structural analyses and electronic properties. (a) XRD pattern of Pt_2_Gd alloy prepared by different annealing temperatures. (b) XANES spectra at the Pt L_3_ edge. The XANES spectrum of Pt_2_Gd-900 at the Pt L_3_ edge is compared with two reference samples of Pt metal foil and Pt/C (inset: image from the partially enlarged view). (c) Detailed comparison of the bond length of Pt. (d) XPS spectrum of the Pt 4f orbit in Pt_2_Gd-900 and Pt/C. (e) XANES spectra at the Gd L_3_ edge. The XANES spectrum of Pt_2_Gd-900 at the Gd L_3_ edge is compared with two reference samples of Gd metal foil and Gd-MOF (inset: image from partially enlarged view). (f) Gd M_4,5_ absorption edge soft XAS spectra for Pt_2_Gd-900 and Gd-MOF.

To verify the composition of the bimetallic alloy, a comprehensive analysis of the individual alloy particles was conducted (Fig. 1d–g). The EDS line scan measurements (Fig. 1d) confirmed the coexistence of Pt and Gd in the same particles. Figure 1f and the inset, representing the Fourier transform pattern of high-resolution transmission electron microscopy (HR-TEM) images, show the (311) and (222) superlattice spots, which is consistent with the X-ray diffraction (XRD) pattern of Pt_2_Gd-900 (Fig. 2a), where the (311) and (222) standard peaks were observed. These results indicate that structure-controlled intermetallic compounds were successfully synthesized. Figure 1g shows a low-magnification, high-angle annular dark-field (HAADF)-STEM image along the ⟨110⟩ direction. A consistent alignment of bright and dark regions representing {111} crystal planes was observed. This indicates two different types of atoms, which is consistent with the simulated atomic structure of the Pt_2_Gd NPs (the image in the inset). Furthermore, a lattice fringe spacing of 2.7 Å was assigned to the {220} planes of the Pt_2_Gd intermetallic compound. Therefore, the combined characterization results unambiguously confirm the successful synthesis of Pt_2_Gd intermetallic compounds via our strategy.

### Structural and electronic characterizations of Pt_2_Gd alloy

The structural and electronic properties of the Pt_2_Gd-900 catalyst were then evaluated (Fig. 2a–f). The XRD patterns in Fig. 2a indicate that the as-synthesized carbon-supported Pt_2_Gd NPs annealed at 900°C, 1000°C and 1100°C show additional (111) and (220) peaks of Pt_2_Gd (the joint committee on powder diffraction standards (JCPDS) 65-1745), which are characteristic peaks of the intermetallic compound structures [39]. The phase transition into the intermetallic phase requires an annealing temperature of at least 900°C, which is consistent with the results of previous studies of Pt-based intermetallic NPs. When the samples were heat treated at higher temperatures, i.e. 1000°C and 1100°C, the peak intensity further increased with a narrow width, indicating an increase in the particle size of the Pt_2_Gd intermetallic NPs [40].

The electronic state of the intermetallic compound NPs is significantly different from that of monometallic or oxidized Pt and Gd. This was confirmed by the normalized X-ray absorption near-edge structure (XANES) analysis at the Pt L_3_ and Gd L_3_ edges (Fig. 2b and e) and the soft X-ray absorption spectroscopy (sXAS) at the Gd M_4,5_ edge (Fig. 2f). The L_3_ edge spectrum of Pt reflects the final state of the electron transition from the 2p_2/3_ state to the 5d_3/2,5/2_ states in Pt. Thus, the weakened white-line intensity relative to Pt/C, which is observed upon the formation of Pt_2_Gd intermetallic compounds, originated from an increase in the Pt 5d electrons or a decrease in the number of d holes [41]. In Fig. 2b, which shows the XANES analysis results of Pt_2_Gd–Pt, the intensity of the white line close to the Pt foil indicates that Pt in Pt_2_Gd is in the metallic state, which excludes the formation of Pt–O bonds in the alloy interface. This result is consistent with the observation of the weakened Pt–O bond intensity of Pt_2_Gd-900 (Fig. 2c). The high-resolution XPS spectra of Pt-4f (Figs 2d and S6) also confirm that most Pt is in a metallic state. The binding energies of the Pt-4f peaks of Pt_2_Gd alloys exhibited a significant negative shift relative to Pt/C. This negative shift in the binding energy can be attributed to the electron charge transfer from Gd to Pt in Pt_2_Gd alloy NPs [3,38].

Next, the 5d state density in the Gd atoms was observed based on the absorption maximum of the L_3_ edge at ∼7245 eV, which corresponds to the 2p_6_4f_7_5d_0_→2p_5_4f_7_5d_1_ transitions. The intensity of the absorption maximum is sensitive to the electronic properties of the surrounding electron acceptors. The XANES spectrum of Pt_2_Gd-900 (Fig. 2e) shows a lower L_3_ edge intensity than that of Gd^3+^ in Gd_2_O_3_ but higher than that of Gd^3+^ in the Gd foil, which demonstrates that the Gd is in a positively charged chemical state (Gd^x+^, 0 < x < 3) in Pt_2_Gd-900 [42]. Considering that the presence of three C KLL Auger transitions located within a binding energy range of 1205–1245 eV could be interfering with feedback (Figs S7 and S8), the measurement of the atomic concentrations of Gd 3d_3/2_ lines in the XPS analysis cannot be performed. Instead, the pre- and post-edge backgrounds of the sXAS spectra were subtracted and normalized at the Gd M_4,5_ absorption edge (Fig. 2f). This method is highly sensitive to the occupancy of the 4f shell, and therefore it enables differentiation between the 4f states of Gd in Pt_2_Gd and Gd-MOF. The intensity of the satellite peaks decreased with the formation of a Pt_2_Gd alloy lattice, indicating greater hybridization strength of the Gd states between the 5d and 4f orbitals of Pt_2_Gd–Gd than that of Gd-MOF [43]. Moreover, no clear difference was observed in the spectral features of Pt_2_Gd and Gd-MOF, and no peak occurred at 1183 eV. Both observations confirmed the absence of any Gd cluster formation [43].

This analysis shows that when Gd components bond with Pt atoms, the Pt-5d orbitals are affected by the d–d orbital hybridization. Moreover, the experimental results show that the 4f states of Gd could affect their electron state during d–d orbital hybridization. These results confirm that intrinsic orbital coupling between Pt and Gd in the alloy definitely induces modulations in the electroactivity of Pt for the ORR.

### In-depth assessment of the shielding effect of spin-reconfigured Pt_2_Gd

**Figure 3.**
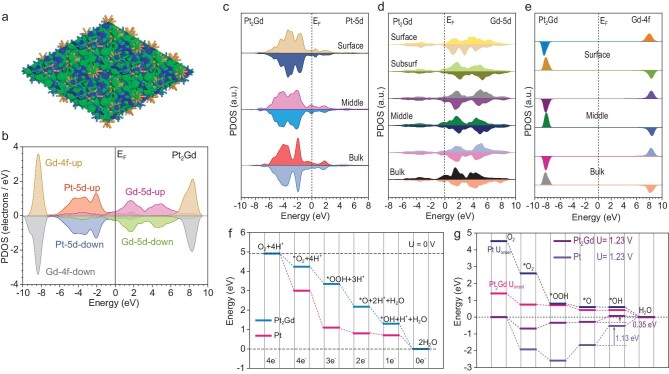
Insight into the spin shielding effect. The 3D contour plot of electronic distribution near the Fermi level of (a) Pt_2_Gd. Dark blue balls = Pt, and orange balls = Gd. Blue isosurface = bonding orbitals and green isosurface = antibonding orbitals. The PDOS of (b) Pt_2_Gd. The site-dependent PDOS of (c) Pt-5d, (d) Gd-5d and (e) Gd-4f in Pt_2_Gd. (f) The reaction energies of the ORR under U = 0 V for Pt_2_Gd and Pt. (g) The reaction energy trends of the ORR under onset potential and equilibrium potentials (U = 1.23 V).

DFT calculations were conducted to investigate the intrinsic oxygen reduction performance of Pt_2_Gd based on the effect of spin reconfiguration on the electronic structures of materials. The electronic distributions near the Fermi level (E_F_) were dominated by both Pt and Gd sites, confirming the modification of electronic structures by the introduction of Gd (Fig. 3a). Compared to the symmetric electronic distributions in Pt, the perturbations induced in the electronic distributions in Pt_2_Gd also enhance the electron transfer between Pt active sites and intermediates [19–22]. Pt_2_Gd exhibits a stable structure in which only slight surface distortion is observed. To reveal spin polarizations, the projected partial densities of states (PDOSs) of both the spin-up and spin-down states were studied (Fig. 3b). Notably, the overall Pt_2_Gd surface displays limited spin polarization due to the presence of highly symmetric states, where only slight PDOS shifting has been noted in Gd-5d and Gd-4f orbitals. The nearly-net-zero spin results in a relatively weak ferromagnetic characteristic. In contrast, for pristine Pt thin film, spin polarizations are more evident in Pt-5d orbitals, particularly near the E_F_ (Fig. S9a). The strongly polarized Pt surface limits the adsorption of polarized intermediates because of the decreased spin selectivity, leading to a lower coverage on the Pt surface. In comparison, the reduced spin polarizations in Pt_2_Gd can improve the adsorption of reactants and intermediates during the ORR. These results indicate that Gd-induced spin polarizations are a pivotal influence on the electroactivity of the material.

To further understand the correlation between spin polarization and electronic structures, the site-dependent PDOSs were further analyzed (Fig. 3c). For the Pt sites in Pt_2_Gd, the symmetry of spin states in the Pt-5d orbitals has been significantly increased. Compared to pristine Pt, the overall Pt-5d orbital in Pt_2_Gd was downshifted, increasing reduction capability of Pt for the ORR. Moreover, Gd sites showed strong spin-polarization effects in both the 5d and 4f orbitals (Fig. 3d). Gd-5d orbitals, throughout the bulk material up to its surface, exhibited asymmetric distributions of the spin-up and spin-down states, which strongly enhanced the magnetic responses of Pt_2_Gd during the experiments. In addition, Gd-4f orbitals formed a ‘reverse spin pair’ in Pt_2_Gd (Fig. 3e). The opposite spins of Gd sites in nearby layers induced a ‘spin shielding effect’ on Gd-4f orbitals, determining the balance of the spin effect in Pt_2_Gd.

To quantify the change in electronic structure, the overall change in the d-band center and PDOS shifting of Pt and Pt_2_Gd were investigated (Fig. S9b). Notably, the d-band center of Pt was downshifted after the introduction of Gd, where the Gd-5d orbitals in a high position forced the Pt-5d orbitals toward a lower position, improving the reduction capability of the catalyst. This further causes an overall increase in the d-band center in Pt_2_Gd to support a more efficient electron transfer, which is consistent with the results illustrated in Fig. 2b, c and e. Polarization effects in the site-dependent PDOSs are also compared, and an efficient electron transfer is observed, which is in good agreement with the results illustrated in Fig. 2b, c and e. In addition, the spin polarization of Pt-5d and Gd-4f significantly decreased (Fig. S9c). The Gd-5d orbitals show an evident shifting in spin states, which complements the spin polarization effect of Pt-5d, leading to the weakening of the spin effect in Pt-5d at the surface. The strong spin-polarized Pt films lower adsorption preferences due to spin repulsive forces with polarized O_2_. When the Pt surface becomes nearly unpolarized, the adsorption of O_2_ becomes stronger, leading to a higher coverage of O_2_ on the Pt surface to promote reaction processes.

From an energetic perspective, the Pt_2_Gd surface modulation decreases the adsorption energy costs of both O_2_ and the proton, indicating improved Pt_2_Gd electroactivity (Fig. S9d). Moreover, the energy change of the ORR process further reveals the different electronic modulations induced by the introduction of Gd into the Pt structure (Fig. 3f). Owing to the alleviation of surface polarization, the initial adsorption of O_2_ shows a strong downhill trend. The smallest energy drop occurs during the conversion from *O to *OH on both Pt_2_Gd and Pt, which is identified as the rate-determining step in the ORR process. At an applied equilibrium potential of 1.23 V, the largest energy barriers in the cases of Pt_2_Gd and Pt were 0.35 and 1.13 eV, respectively (Fig. 3g). The much smaller energy barrier of Pt_2_Gd increases onset potential, which reflects the superior ORR performance of Pt_2_Gd. These results have revealed that Gd-induced electronic modulations are critical to realizing improved electrocatalysis.

### Spin-polarized kinetics of the ORR

**Figure 4.**
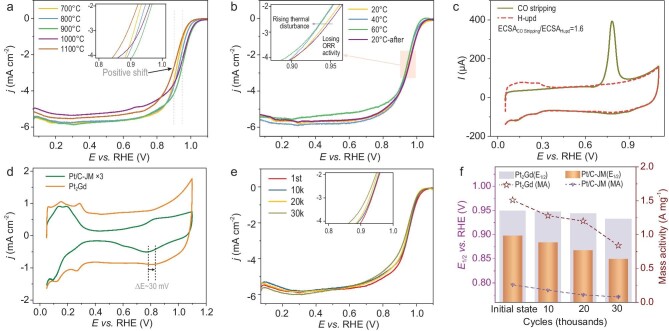
Catalytic performance. (a) Comparison of the ORR activity of Pt_2_Gd-X (X = 700, 800, 900, 1000, 1100) in LSV curves (inset: contrast of E_1/2_ from partially enlarged view). (b) LSV curves of temperature control in the ORR (inset: contrast of E_1/2_ from partially enlarged view). (c) CO stripping and H_upd_ test for structure confirmation. (d) CV curves in N_2_ atmosphere of Pt_2_Gd-900 and Pt/C. (e) LSV curves of Pt_2_Gd-900 before and after 10k, 20k, 30k cycling durability tests at 25°C in 0.1 M HClO_4_ electrolyte (inset: contrast of E_1/2_ from partially enlarged view). (f) Comparison of mass activities (MAs) at 0.9 V and E_1/2_ versus reversible hydrogen electrode (RHE).

The ORR activity of the as-prepared Pt_2_Gd alloy was then evaluated using a rotating disk electrode in a 0.1 M HClO_4_ electrolyte (Fig. 4). Here, the oxygen reduction curves (obtained by linear sweep voltammetry, LSV) of Pt_2_Gd alloys annealed at different temperatures were measured (supplementary materials). Controlling the treatment temperature is considered an equivalent scheme for introducing the spin effect to the local electronic state via the alloying procedure [44,45]. Pt_2_Gd-900 exhibited a high half-wave potential (E_1/2_) of 0.95 V versus a reversible hydrogen electrode, with a well-defined diffusion limiting current (Fig. 4a). The catalytic performance of the alloy increased with the increase in heat treatment temperature from 700°C to 900°C, which can be attributed to a higher intermetallic ordering degree in the final samples. However, with a further increase in temperature, above 1000°C, an increase in particle size is observed, and hence Pt utilization decreases, reducing overall performance. These results are consistent with the XRD patterns.

To study the possible spin effect of Gd on the final catalytic behavior of Pt, the ORR performance of Pt_2_Gd-900 was tested at different temperatures to introduce thermal disturbance to the spintronic characteristic of Pt, which consequently reflects on ORR performance. Typically, thermal intervention is an effective method for regulating the magnetic microstructure or magnetic domains. As the thermal disturbance increases, electrons with spin move in a disordered manner, decreasing local intrinsic magnetism. Thus, the magnetic moment along the magnetic field direction of the sites of each magnetic unit changes, and the strength of the magnetic attraction of the material to the external interface also changes. As a result, increasing the treatment temperature weakens the magnetic moment component, and decreasing the temperature restores it (Fig. S10) [44]. In our experimental observations, the difference (ΔM) between the field-cooling and zero-field-cooling curves has proven that the intrinsic spin state of the Pt_2_Gd alloy begins to weaken with an increase in temperature from room temperature to 400 K. Thus, the ORR was subjected to thermal disturbance in order to observe the corresponding change in the ORR performance of the intrinsically spin-reconfigured Pt_2_Gd alloy. The onset potential and pre-catalytic basal current were identical in these regimes. However, the E_1/2_ of Pt_2_Gd-900 shows a mild negative shift with an increase in reaction temperature from 20°C to 40°C and then to 60°C (Fig. 4b). Thus, the shielding effect has been weakened by the gradual increase in thermal disturbance, thereby decreasing ORR performance. Therefore, when the testing temperature was reset to 20°C, ORR performance recovered to its initial state (inset of Fig. 4b). This can be attributed to the rearrangement of single electrons into ordered states and the intrinsic spin recovery after removing thermal perturbation. Based on the DFT calculations reported in Fig. 4d, the oxide reduction peak potential in the Pt_2_Gd alloy increased by ∼30 mV, showing that the desorption of OH at the reaction interface is favorable in this case compared with the pure Pt case.

The stabilities of the Pt_2_Gd-900 catalyst and the commercial benchmark Pt/C catalyst were then compared, revealing that Pt_2_Gd-900 is even more stable than Pt/C. The electrochemically active surface area (ECSA) of Pt_2_Gd (Fig. 4c) was estimated. The ratio of ECSA that was measured according to the CO stripping method (ECSA-CO stripping), and the ratio of ECSA that was estimated according to the hydrogen desorption charge (ECSA-Hupd), was 1.6. This indicates that the catalyst surface is Pt-rich and structurally stable because the adsorption strength of hydrogen on the Pt-skin surface is low, which is consistent with Figs 1g, S7 and S8 [3]. After 30k cycles of the accelerated stability test (AST), the half-wave potential of the catalyst only negatively shifted by ∼17 mV (Fig. 4e). The HR-TEM images before and after AST were compared, and no significant aggregation of particles was observed (Figs S11 and S12). Thus, no significant loss was observed in ECSA (Fig. S13). Mass activities were obtained by normalizing kinetic currents with the Pt loading of the corresponding catalysts (Fig. 4f). Pt_2_Gd-900 shows an initial mass activity of 1.51 A·mg_Pt_^−1^, which is five times that of the Pt/C commercial catalyst (0.26 A·mg_Pt_^−1^). After 30k cycles, only 30% of the mass activity of the Pt/C-JM was preserved, whereas 60% of that of Pt_2_Gd alloy was preserved. In our next study, we will explore a technique towards suppressing Pt dissolution (Table S1) to further improve the stability of the Pt-based alloy catalyst.

## CONCLUSION

**Scheme 1.**
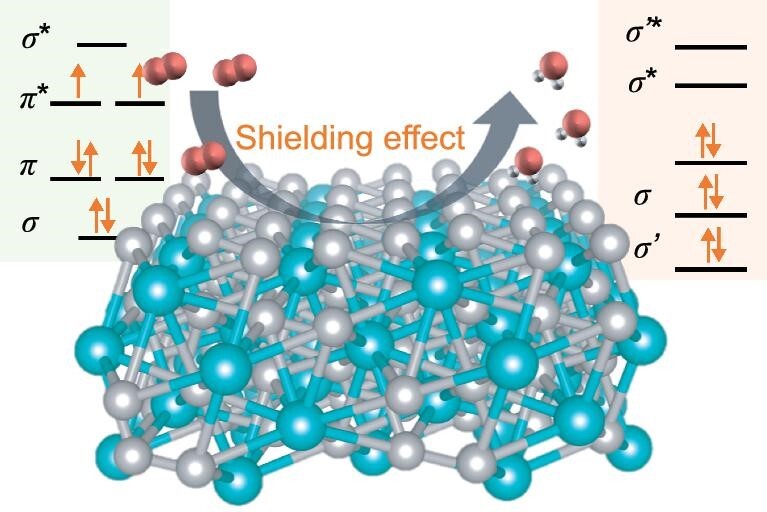
Schematic diagram of the spin shielding effect in oxygen electrocatalysis.

A simple method that utilizes the unique structure of MOFs was proposed for the synthesis of Pt–rare-earth-metal-based intermetallic alloys. The proposed method can easily overcome the chemical potential difference between single-atom rare-earth elements and Pt. In addition, ORR dependence on the spin states of the material has been experimentally proven for the first time (Scheme 1). In an acidic electrolyte environment, the activity and stability of the Pt_2_Gd alloy with an intrinsic shielding effect were significantly improved, exhibiting a high half-wave potential of 0.95 V and a mass activity of 1.5 A·mg_Pt_^−1^. DFT calculations demonstrated that the electroactivity of Pt-5d orbitals in Pt_2_Gd was optimized through the complementary spin polarization of Pt and Gd. The Gd sites induced a spin shielding effect, which balanced the spin polarizations of the Pt-5d orbitals and improved the reduction capability of Pt sites. The spin reconfigurations in Pt_2_Gd have optimized the electronic structures of Pt and improved adsorption of the oxygen intermediates, ensuring high ORR performance. This work provides significant contributions with regard to enhancing the understanding of the spin-modulation effect on electrocatalytic performance of materials. Considering the limited amount of Gd on Earth, it is also crucial to scale up the proposed performance-enhancing modulation method for use in practical devices and applications with more abundant elements. We have now verified the validity of the synthesis method for Pt_2_Gd alloys in many rare-earth elements, such as La, Nd, Er, Lu, etc., and this will be discussed in our future studies.

## METHODS

### Synthesis of MOF-76(Gd)

MOF-76(Gd) was synthesized via a solvothermal synthesis method. 1,3,5-benzenetricarboxylic acid (BTC, 0.428 g) and GdCl_3_·6H_2_O (0.577 g) were dissolved in a cosolvent (100 mL) composed of 1 : 1 water and N, N-dimethylformamide (DMF) under stirring. The mixture was then stirred for 3 h at 90°C before being dried for 12 h at 60°C. The obtained fluffy white powder was carbonized at 900°C for 1 h at a heating rate of 5°C min^−1^ in an Ar gas atmosphere in a tubular carbonization furnace. The obtained sample is designated MOF-76(Gd).

### Synthesis of Pt_2_Gd-900 alloy

Pt_2_Gd-900 alloy was manufactured using a wet chemical electrostatic adsorption method. MOF-76(Gd) (100 mg) and H_2_PtCl_6_·H_2_O (3.7 mg) were simultaneously added to secondary deionized water (30 ml), stirred for 12 h, and then evaporated and dried by a rotary evaporation apparatus. The obtained fluffy black powder was carbonized at 900°C for 2 h at a heating rate of 5°C min^−1^ in a 5% H_2_ gas atmosphere in a tubular carbonization furnace. The product was dried after 12 h of stirring in a 0.1-M HClO_4_ solution. The obtained sample is designated Pt_2_Gd-900. Pt_2_Gd-700, Pt_2_Gd-800, Pt_2_Gd-1000 and Pt_2_Gd-1100 were prepared using a similar process to that of Pt_2_Gd-900 but at different pyrolysis temperatures.

## Supplementary Material

nwad162_Supplemental_File
