# Supplementary material for: Intrinsic spin shielding effect in platinum–rare-earth alloy boosts oxygen reduction activity
Source: Natl Sci Rev. 2023 Jun 9;10(9):nwad162. doi: 10.1093/nsr/nwad162 (PMC10600901; doi:10.1093/nsr/nwad162)
Supplement: nwad162_Supplemental_File [file nwad162_supplemental_file.docx]

**Supplementary Information**

**Intrinsic Spin Shielding Effect in Platinum–Rare Earth Alloy Boosts Oxygen Reduction Activity**

Siyuan Zhu^1,2†^, Mingzi Sun^3†^, Bingbao Mei^4^, Liting Yang^1,2^, Yuyi Chu^1,2^, Zhaoping Shi^1,2^, Jingsen Bai^1,2^, Xian Wang^1,2^, Zheng Jiang^4^, Changpeng Liu^1,2^, Bolong Huang^3^*, Junjie Ge^1,2,5^*, Wei Xing^1,2^*

† The authors contribute equally.

^1^ State Key Laboratory of Electroanalytical Chemistry, Laboratory of Advanced Power Sources, Changchun Institute of Applied Chemistry, Chinese Academy of Sciences, Changchun 130022, China.

^2^ School of Applied Chemistry and Engineering, University of Science and Technology of China, USTC, Hefei 230026, China.

^3^ Department of Applied Biology and Chemical Technology, The Hong Kong Polytechnic University, Hung Hom, Kowloon, Hong Kong SAR 999077, China.

^4^ Shanghai Synchrotron Radiation Facility, Zhangjiang National Laboratory, Shanghai Advanced Research Institute, Chinese Academy of Sciences, Shanghai 201204, China.

^5^ Dalian National Laboratory for Clean Energy, Chinese Academy of Sciences, Dalian 116023, China.

* Corresponding authors.

E-mail: xingwei@ciac.ac.cn;

gejunjie@ustc.edu.cn;

bhuang@polyu.edu.hk

1. **Extended Methods**

**Characterizations**

Scanning electron microscopy (SEM) measurements were performed with an XL 30 ESEM FEG field emission scanning electron microscope. Transmission electron microscopy (TEM), high resolution transmission electron microscopy (HRTEM), high-annular dark-field scanning transmission electron microscopy (STEM) and element mapping analysis were conducted on Philips TECNAI G2 electron microscope operating at 200 kV. X-ray photoelectron spectroscopy (XPS) measurements were carried out on Mg Kα radiation source (Kratos XSAM-800 spectrometer). The bulk compositions were evaluated by inductively coupled plasma optical emission spectrometer (X Series 2, Thermo Scientific USA).

**Electrocatalytic measurements**

All electrochemical measurements were conducted in a conventional three-electrode cell at room temperature (~25 °C) using the 750E Bipotentiostat (CH Instruments). The Pt_2_Gd-900 catalyst ink and Pt/C catalyst (20 wt% Pt on Vulcan XC-72 carbon, Johnson Matthey) ink were both prepared by ultrasonically dispersing 5 mg catalyst in a suspension containing 50 μL Nafion (5 wt%) solution and 550 μL isopropanol and 400 μL Milli-Q water. The catalyst film coated electrode was obtained by dispersing the catalyst ink on a glassy carbon rotating ring-disk electrode followed by drying in air. The catalyst loadings on RRDE were 10 μg_Pt_ cm^-2^ for Pt_2_Gd-900 catalyst and 20 μg_Pt_ cm^-2^ for commercial Pt/C catalyst. RDE measurements were conducted by linear sweep voltammetry (LSV) from 1.1 V to 0.05 V at a scan rate of 10 mV s^-1^ at 1600 rpm. The ORR stability was investigated by continuous potential cycling in oxygen-saturated 0.1 M HClO_4_ solution between 0.6 V and 1.05 V with the scan rate at 100 mV s^-1^. And after 30,000 cycles, the ORR steady-state polarization measurements were conducted in O_2_-saturated 0.1 M HClO_4_ solution with scanning rates of 10 mV/s and rotation rate at 1600 rpm. All the ORR currents presented in the figures are faradaic currents, i.e. after correction for the capacitive current and have already been normalized to the electrode surface area.

The following equations were used to calculate n (the apparent number of electrons transferred during the ORR) and % H_2_O_2_ (the percentage of H_2_O_2_ released during the ORR).

$$\text{n=}\frac{\text{4}\text{I}_{\text{D}}}{\text{I}_{\text{D}}\text{+}{\text{I}_{\text{R}}}/\text{N}}\text{ (1)}$$

$$\text{\%}\text{H}_{\text{2}}\text{O}_{\text{2}}\text{=}\frac{\text{200}{{\text{I}\text{ }}_{\text{R}}}/\text{N}}{\text{I}_{\text{D}}\text{+}{\text{I}_{\text{R}}}/\text{N}}\text{ (2)}$$

where *I*_D_ is the faradaic current at the disk, *I*_R_ is the faradaic current at the ring and *N* is the collection coefficient at the ring. The apparent number of electrons transferred for the ORR was also determined by the Koutecky–Levich equation given blow:

$$\frac{\text{1}}{\text{J}}\text{=}\frac{\text{1}}{\text{J}_{\text{L}}}\text{+}\frac{\text{1}}{\text{J}_{\text{K}}}\text{=}\frac{\text{1}}{\text{Bω}^{\text{1/2}}}\text{+}\frac{\text{1}}{\text{J}_{\text{k}}}\text{ (3)}$$

$$\text{B=0.62}\text{nF}\text{C}_{\text{0}}{\text{(}\text{D}_{\text{0}}\text{)}}^{\text{2/3}}\text{ν}^{\text{1/6}}\text{ (4)}$$

where *J* is the measured current density, *J*_K_ is the kinetic current density, *J*_L_ is the diffusion-limited current density, *ω* is the electrode rotation rate, *F* is Faraday constant (96485 C mol^-1^), *C*_0_ is the bulk concentration of O_2_ (1.2×10^-3^ mol L^-1^ for 0.1 M HClO_4_ solution), *D*_0_ is the diffusion coefficient of O_2_ (1.93×10^-5^ cm^2^ s^-1^ for 0.1 M HClO_4_ solution and *ν* is the kinetic viscosity of the electrolyte (0.01 cm^2^ s^-1^ for both 0.1 M HClO_4_ solution). The formulas for calculating the electrochemical surface area (denoted as S) and atomic utilization ratio (denoted as A) are as follows:

$$\text{S}\text{ =}\frac{\text{Q}\text{(}\text{μA}\text{ }\text{· mV)}}{\text{10 }\text{m}V/s\text{× }\text{210 }\text{μC}\text{ / cm}\text{2}}\text{ }\text{ (5)}$$

$$\text{S}\text{ =}\frac{\text{Q}\text{(}\text{μA}\text{ }\text{· mV)}}{\text{10 }\text{m}V/s\text{× }\text{420 }\text{μC}\text{ / cm}\text{2}}\text{ (6)}$$

$$\text{A}\text{ =}\frac{\text{Q}\text{CO}\text{(}\text{μA}\text{ }\text{·mV)}\text{/}\text{e}}{\text{ }\text{m}/\text{NA}}\text{ (7)}$$

where (5) is for H-upd method and (6) is for CO stripping method, Q_co_ is quantity of electricity provided by monolayer CO adsorbed on the platinum surface, e is 1.6021892×10 C, m is loading of metal on electrode, and NA is 6.02214076×10²³.

**Calculation Setup**

To study the electronic structures of Pt_2_Gd regarding spin polarization, DFT calculations based on the CASTEP packages have been used in this work [1]. To supply accurate descriptions of the exchange-correlation interactions, the generalized gradient approximation (GGA) and Perdew-Burke-Ernzerhof (PBE) functionals have been applied [2-4]. Based on the ultrasoft pseudopotentials, the plane-wave basis cutoff energy has been set to 380 eV for the geometry optimizations. The Broyden-Fletcher-Goldfarb-Shannon (BFGS) algorithm has been used for energy minimizations [5]. To balance the calculation efficiency and accuracy, we have selected the coarse quality of k-points for the geometry optimizations. In this work, we have carried out the spin-polarized DFT calculations with CASTEP. The initial magnetic configuration is defined by specifying the total magnetic moment per unit cell, which gets uniformly distributed over the space. The initial magnetic moments on atoms are treated correctly by CASTEP with the density mixing scheme for electronic structure minimization. The value of the total moment is determined by the sum of formal spins in the system. Meanwhile, we have also included a sufficient number of empty bands when optimizing the spin state. The surfaces of both Pt and Pt_2_Gd have been built with six-layer thickness, where all layers are not fixed during the relaxation. The strict convergence criteria have been set for the Hellmann-Feynman forces, the total energy difference, and the inter-ionic displacement, which should not exceed 0.001 eV/Å, 5×10^-5^ eV/atom, and 0.005 Å, respectively.

1. Clark SJ, Segall MD, Pickard CJ, Hasnip PJ, Probert MJ, Refson K, Payne MC. First Principles Methods Using Castep. *Zeitschrift Fur Kristallographie* 2005; **220**: 567-570.
2. Perdew JP, Burke K, Ernzerhof M. Generalized Gradient Approximation Made Simple. *Phys. Rev. Lett*. 1996; **77**: 3865-3868.
3. Hasnip PJ, Pickard CJ, Electronic Energy Minimisation with Ultrasoft Pseudopotentials. *Comput. Phys. Commun.* 2006; **174**: 24-29.
4. Perdew JP, Chevary JA, Vosko SH, Jackson KA, Pederson MR, Singh DJ, Fiolhais C. Atoms, Molecules, Solids, and Surfaces: Applications of the Generalized Gradient Approximation for Exchange and Correlation. *Phys. Rev. B.* 1992; **46**: 6671-6687.
5. Head JD, Zerner MC. A Broyden Fletcher Goldfarb Shanno Optimization Procedure for Molecular Geometries. *Chem. Phys. Lett.* 1985; **122**: 264-270.
6. **Supplemental Figures and Tables**


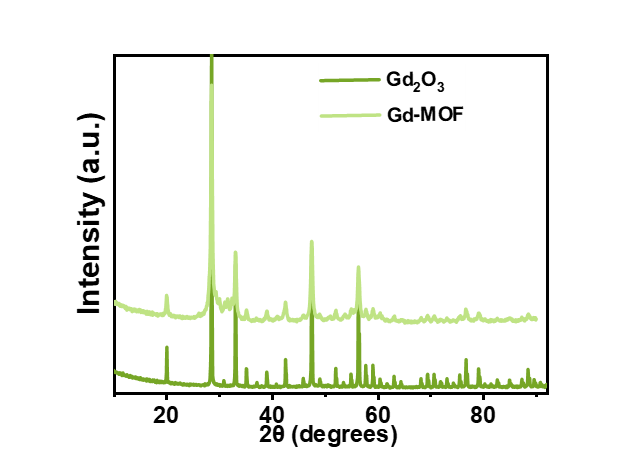


Fig. S1 Comparison of XRD patterns between Gd-MOF and Gd_2_O_3_.


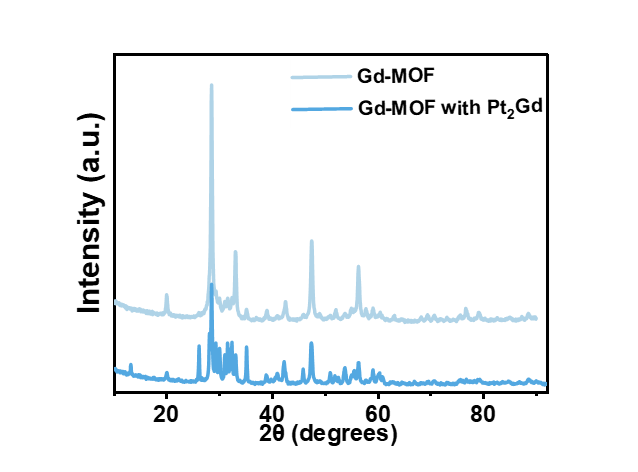


Fig. S2 Comparison of XRD patterns between Gd-MOF and Gd-MOF with Pt_2_Gd before acid washing.

**
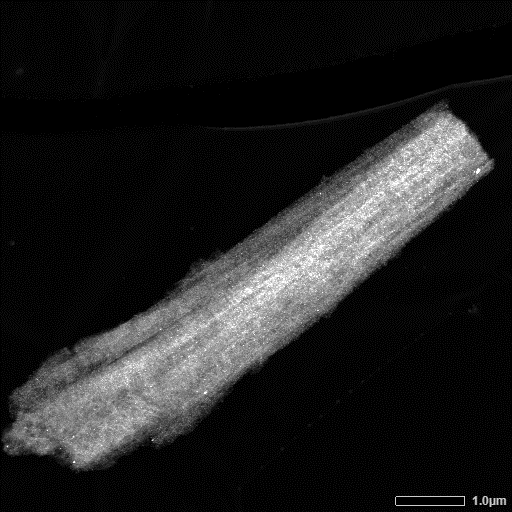
**

Fig. S3 Annular dark-field STEM image of the Pt_2_Gd-900 supported on the Gd-MOF in 1μm scale.


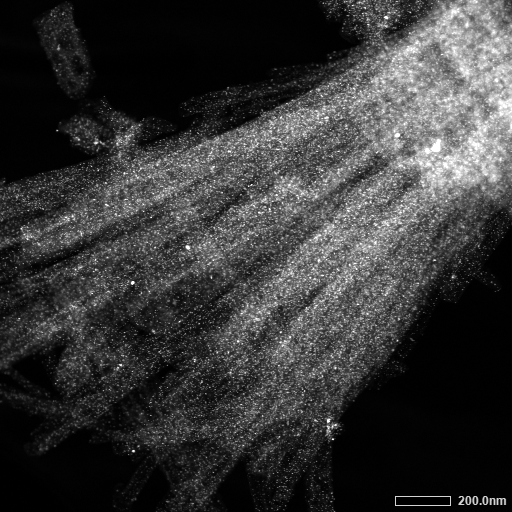


Fig. S4 Annular dark-field STEM image of the Pt_2_Gd-900 supported on the forked Gd-MOF in 200 nm scale.


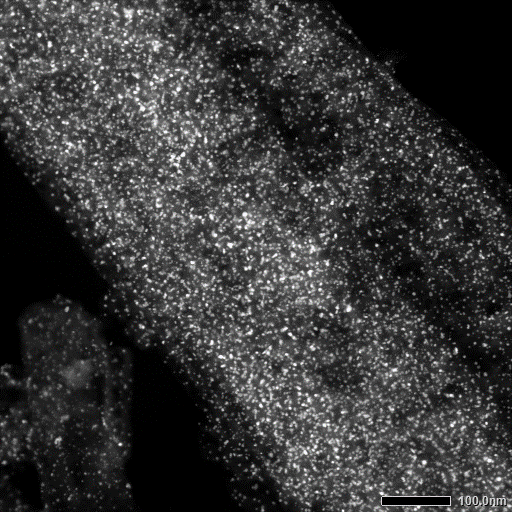


Fig. S5 Annular dark-field STEM image of the Pt_2_Gd-900 supported on the Gd-MOF in 100 nm scale.


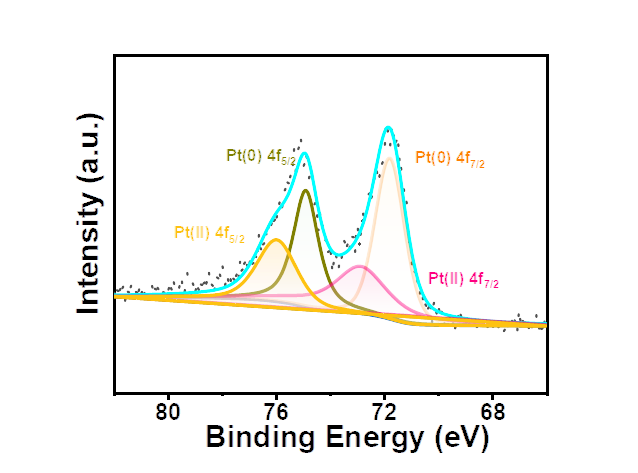


Fig. S6 Deconvoluted XPS spectra of Pt-4f in Pt_2_Gd-900.


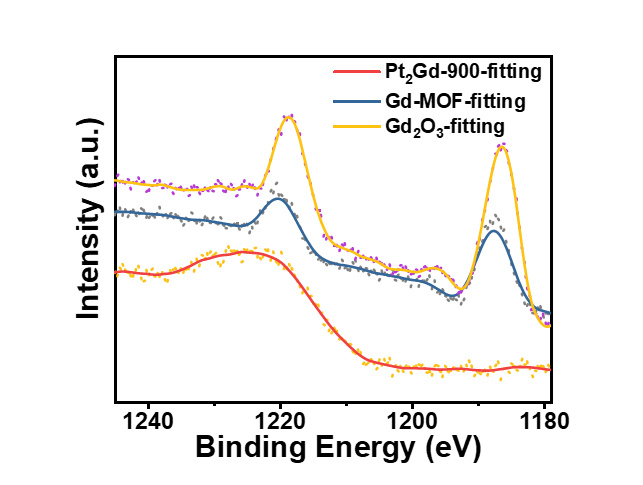


Fig. S7 XPS spectra of Gd-3d in Pt_2_Gd-900,Gd-MOF and Gd_2_O_3_.


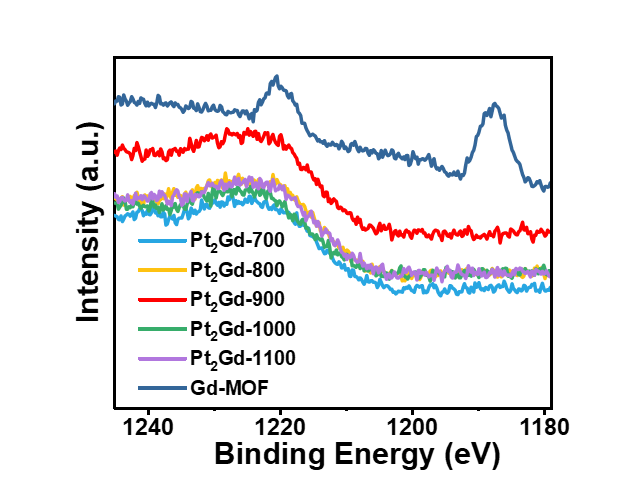


Fig. S8 XPS spectra of Gd-3d in Pt_2_Gd-x and Gd-MOF.


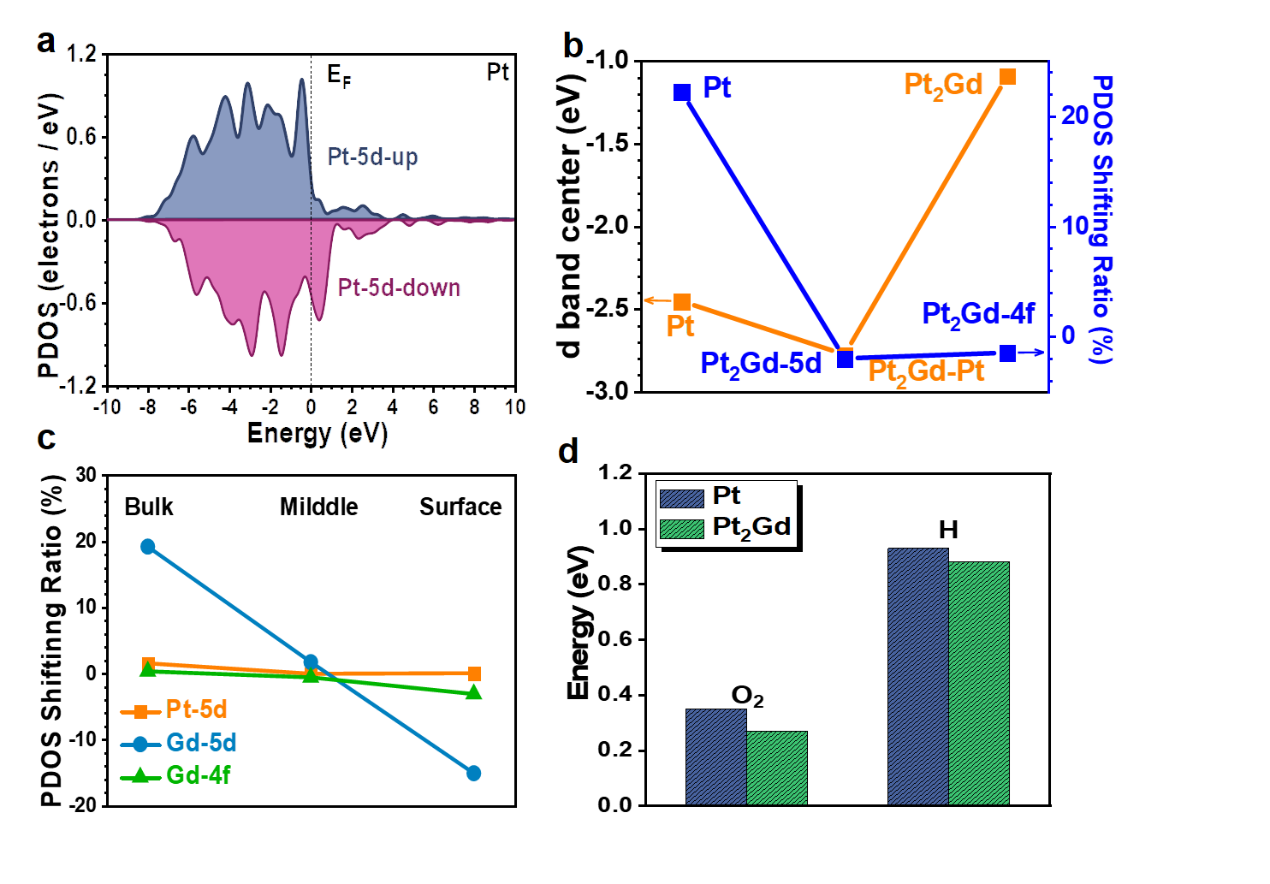


Fig. S9 The PDOS of (a) Pt. (b) The comparison of d-band center and PDOS shifting in Pt_2_Gd and Pt. (c) The site-dependent PDOS shifting comparisons in Pt_2_Gd. (d) The adsorption energies of O_2_ and proton on Pt_2_Gd and Pt.


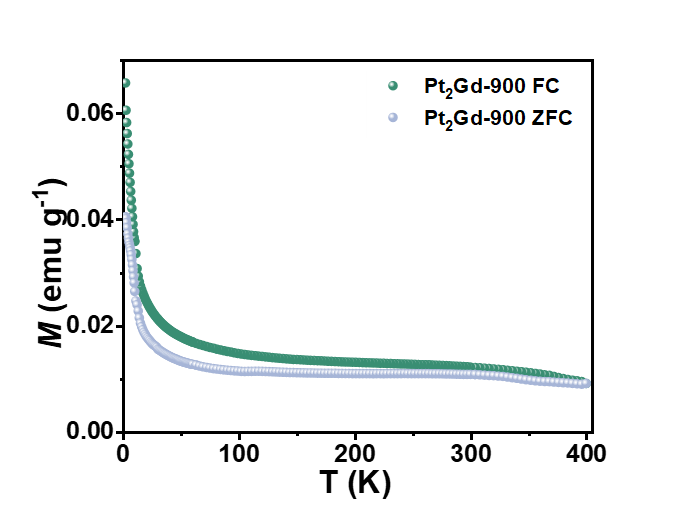


Fig. S10 FC and ZFC curves of Pt_2_Gd-900.


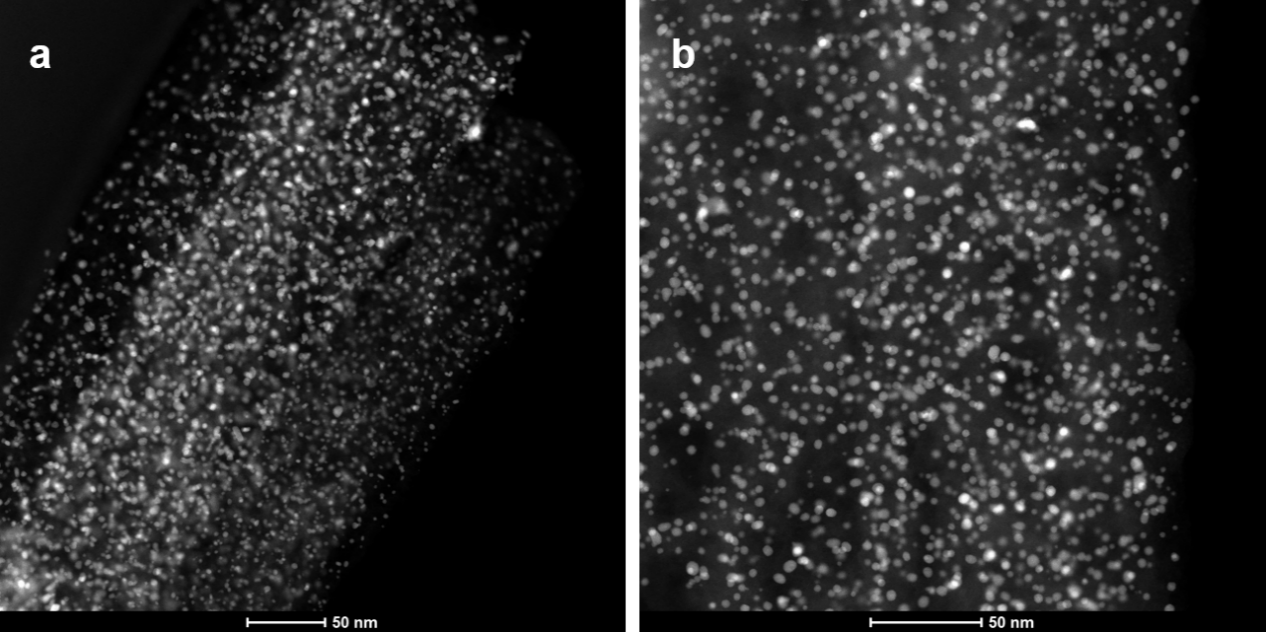


Fig. S11 HADDF-STEM images of the Pt_2_Gd-900 a) before and b) after 30k AST in 50 nm scale.


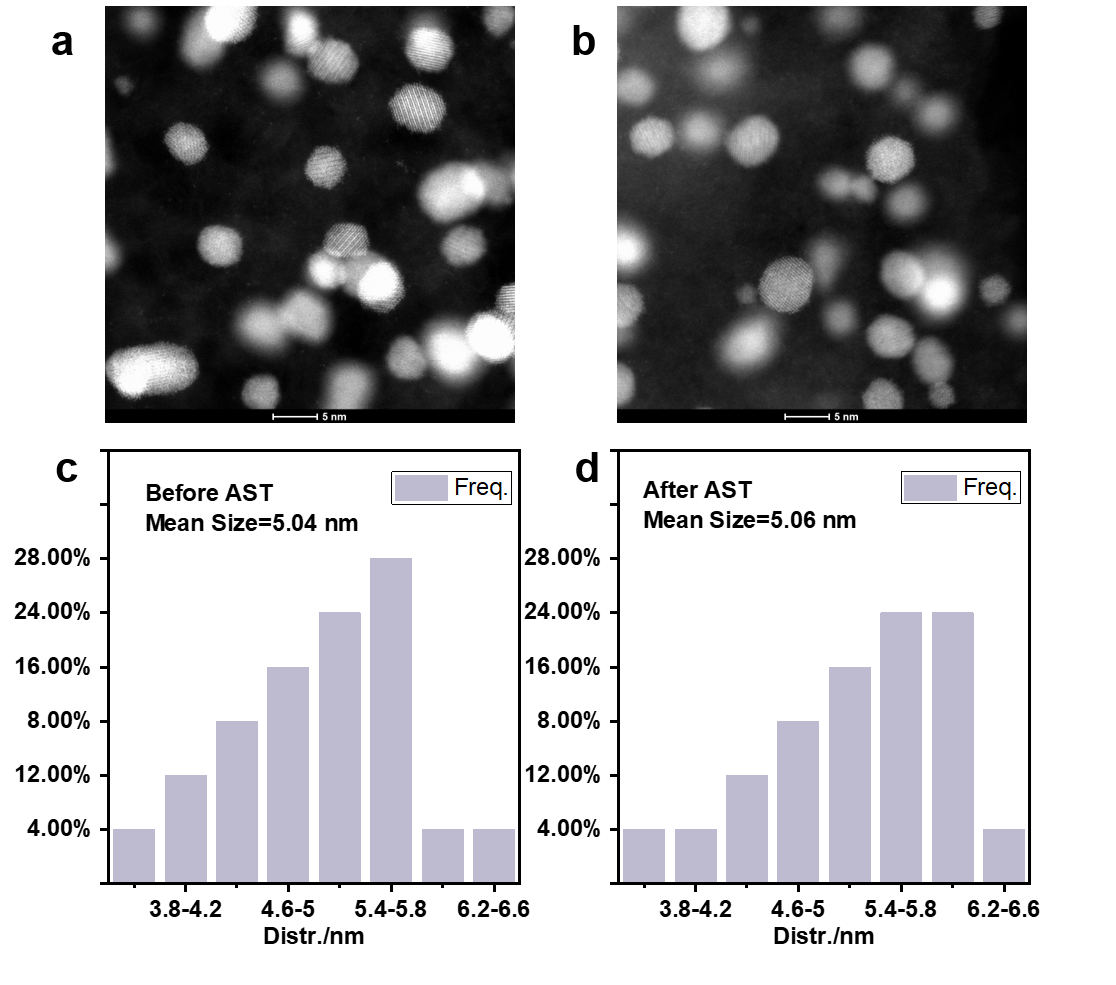


Fig. S12 HADDF-STEM images and size distribution measurements of the Pt_2_Gd-900 a, c) before and b, d) after 30k AST in 5 nm scale.


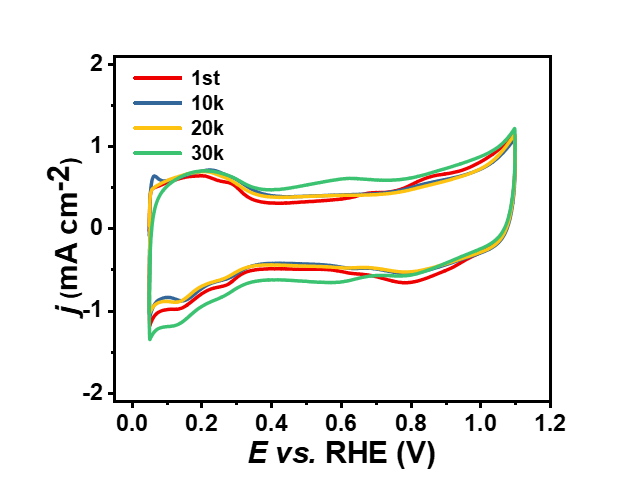


Fig. S13 CV curves before and after 10k, 20k, 30k cycling durability tests at 25℃ in 0.1 M HClO_4_ electrolyte.

Table S1 Element content measurement of Pt and Gd in the collected solution after different AST cycles by ICP-MS (unit: ppb).

| AST cycles | Pt/ppb | Gd/ppb |
| --- | --- | --- |
| 1st | BDL | BDL |
| 5k | BDL | 0.469 |
| 10k | BDL | BDL |
| 20k | BDL | BDL |
| 30k | 0.897 | BDL |

BDL=Below the detection limit.
